# Microporous Biodegradable Films Promote Therapeutic Angiogenesis

**DOI:** 10.1002/adhm.202000806

**Published:** 2020-07-14

**Authors:** Eseelle K. Hendow, Mehran Moazen, Francesco Iacoviello, Laurent Bozec, Caroline Pellet‐Many, Richard M. Day

**Affiliations:** ^1^ Centre for Precision Healthcare UCL Division of Medicine University College London Gower Street London WC1E 6BT UK; ^2^ UCL Mechanical Engineering University College London Torrington Place London WC1E 7JE UK; ^3^ Electrochemical Innovation Lab UCL Department of Chemical Engineering University College London Roberts Building London WC1E 7JE UK; ^4^ Faculty of Dentistry University of Toronto 124 Edwards Street Toronto Ontario M5G 1G6 Canada; ^5^ Department of Comparative Biomedical Sciences Royal Veterinary College 4 Royal College Street London NW1 0TU UK

**Keywords:** poly(d,l‐lactide‐*co*‐glycolide), therapeutic angiogenesis, thermally induced phase separation

## Abstract

Peripheral arterial disease and critical limb ischemia are common symptoms of cardiovascular disease. Vascular surgery is used to create a bypass around occluded blood vessels to improve blood flow to ischemic muscle, thus avoiding the need for amputation. Attempts to vascularize tissues by therapeutic angiogenesis using delivery of exogenous angiogenic agents are underwhelming. A material‐based approach that provides an endogenous stimulus capable of promoting angiogenesis and increased tissue perfusion would provide a paradigm shift in treatment options available. It is reported here that microporous biodegradable films produced using thermally induced phase separation provide a localized biophysical stimulus of proangiogenic genes in vivo that is associated with increased blood vessel density and restoration of blood flow to ischemic tissue. These findings show, for the first time, that acellular, nonfunctionalized biodegradable biomaterials can provide an innovative, material‐based approach for therapeutic angiogenesis to enhance tissue reperfusion in vivo.

## Graphical abstract

Microporous biodegradable film produced using thermally induced phase separation is developed to promote therapeutic angiogenesis for cardiovascular disease. The film stimulates restoration of vascular perfusion in ischemic tissue via upregulation of proangiogenic growth genes that result in increased vessel in density. The new material has potential transform treatment of cardiovascular disease.

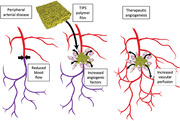

## 1 Introduction

Peripheral arterial disease (PAD) is often caused by the build‐up of atherosclerotic fat in the arteries, which, if left untreated, can develop into limb ischemia and other forms of cardiovascular disease.^[^ ^1^ ^]^ PAD is becoming more prevalent with over 200 million people affected worldwide as a result of an ageing population and an increase in obesity.^[^ ^2^ ^]^ Currently there is no curative treatment for PAD. Therefore, there is a clear need for new therapeutic strategies to promote angiogenesis capable of vascularizing ischemic tissue.

Researchers have strived to achieve improved vascularization of ischemic tissue using strategies that involve delivery of angiogenic growth factors, cell therapy, and gene therapy. Preclinical studies investigating the delivery of angiogenic growth factors, such as vascular endothelial growth factor (VEGF), platelet‐derived growth factor (PDGF), and fibroblast growth factor (FGF), to increase vascularization have been encouraging but these results have not been translated into clinical efficacy.^[^ ^3^ ^]^ Reasons for the lack of stabilization and maturation of newly formed blood vessels, vital for clinical efficacy, include diffusion of the therapeutic agent from the target site,^[^ ^4^ ^]^ or rapid degradation of peptide growth factors rendering them ineffective (for example, VEGF has a half‐life in vivo of ≈30 min).^[^ ^5^ ^]^ Gene therapy has been explored for sustained release of angiogenic factors in patients with PAD;^[^ ^6^ ^]^ however, concerns have been raised regarding the risk of oncogenic effects with this approach.^[^ ^7^ ^]^ Moreover, delivery of proangiogenic agents, either as proteins or genes, in the form of monotherapy does not replicate the plethora of factors that underlie the physiological process of angiogenesis in vivo, where a complex interplay of factors from different cell types is required to form stable blood vessels.^[^ ^8^ ^]^ Alternative approaches involving delivery of cells capable of providing a proangiogenic secretome are faced with similar challenges, including cell retention and survival at the implant site.^[^ ^9^, ^10^, ^11^ ^]^ Attempts to refine the delivery process using combination products, consisting of materials combined with angiogenic agents, to provide more targeted and sustained modes of delivery have achieved only limited success.^[^ ^12^, ^13^, ^14^ ^]^

A more robust therapeutic response might be achieved by identifying stimuli capable of triggering a biological response that results in the plethora of angiogenic factors capable of achieving functional tissue vascularization. We propose this approach could be achieved using synthetic materials exhibiting optimal structural and mechanical properties. Evidence to support the feasibility of this approach exists from previous work that has indicated biophysical cues controlling features of the local microenvironment, such as defined stiffness^[^ ^15^ ^]^ and material topography,^[^ ^16^ ^]^ are important regulators of cell behavior, influencing cell proliferation and differentiation,^[^ ^17^ ^]^ macrophage fusion,^[^ ^18^ ^]^ and secretion of soluble cytokines.^[^ ^19^ ^]^ However, to date there is a paucity of data demonstrating translation of these effects in physiological in vivo environments and/or evidence of therapeutic angiogenesis in ischemic tissue. Recently, activation of angiogenesis and formation of microvasculature in vivo have been reported with injectable amorphous hydrogels exhibiting varying stiffness.^[^ ^20^ ^]^ Similarly, amorphous materials consisting of natural polymer gels, including fibrin and extracellular matrix (ECM)‐derived hydrogels, as well as highly porous gelatin films, have been reported to result in processes associated with angiogenesis.^[^ ^12^, ^21^, ^22^, ^23^ ^]^ Taken together these results suggest mechanically defined microenvironments created by implanting materials could be exploited to deliver local stimuli that translate into proangiogenic signals, providing new approaches for delivering therapeutic angiogenesis.

Here we show, for the first time, that the topographic features of a biodegradable material can be utilized to achieve therapeutic angiogenesis capable of restoring perfusion of ischemic tissue in vivo via stimulation of endogenous angiogenic factors that result in increased tissue vascularization. In this pilot study, the effect is achieved using the biodegradable synthetic polyester material, poly(d,l‐lactide‐*co*‐glycolide) (PLGA), widely used in existing medical devices, such as meshes and sutures, that have been processed via thermally induced phase separation (TIPS) to create micropored topographical features. We have previously demonstrated that TIPS‐processed materials with other geometries provide an excellent substrate for cell attachment in vitro and become rapidly with host tissue when implanted subcutaneously into soft tissues.^[^ ^24^, ^25^ ^]^ However, to achieve an efficacious therapeutic angiogenic effect, the response associated with the material needs to provide sufficient neovascularization capable of restoring tissue perfusion. In order to evaluate the level of angiogenic potency, the current study used multiple methods to investigate the in vivo angiogenic effect of TIPS‐processed PLGA polymer films in ischemic tissue.

## 2 Results and Discussion

TIPS is a widely used technique for the fabrication of 3D polymeric tissue scaffolds with a range of geometries.^[^ ^26^, ^27^ ^]^ TIPS has also been used extensively for the preparation of porous membranes and films from a variety of polymers, including poly(vinylidene fluoride) (PVDF), polyethylene, polypropylene, poly(methyl methacrylate), polystyrene, and poly(ethylene‐*co*‐vinyl alcohol),^[^ ^28^, ^29^, ^30^, ^31^, ^32^, ^33^ ^]^ for applications including filtration, quasi‐solid electrolytes for electrical devices, and dermal tissue engineering scaffolds.^[^ ^34^, ^35^ ^]^ The current study is the first to investigate the use of TIPS films composed of PLGA specifically for use in therapeutic angiogenesis.

**Figure 1:**
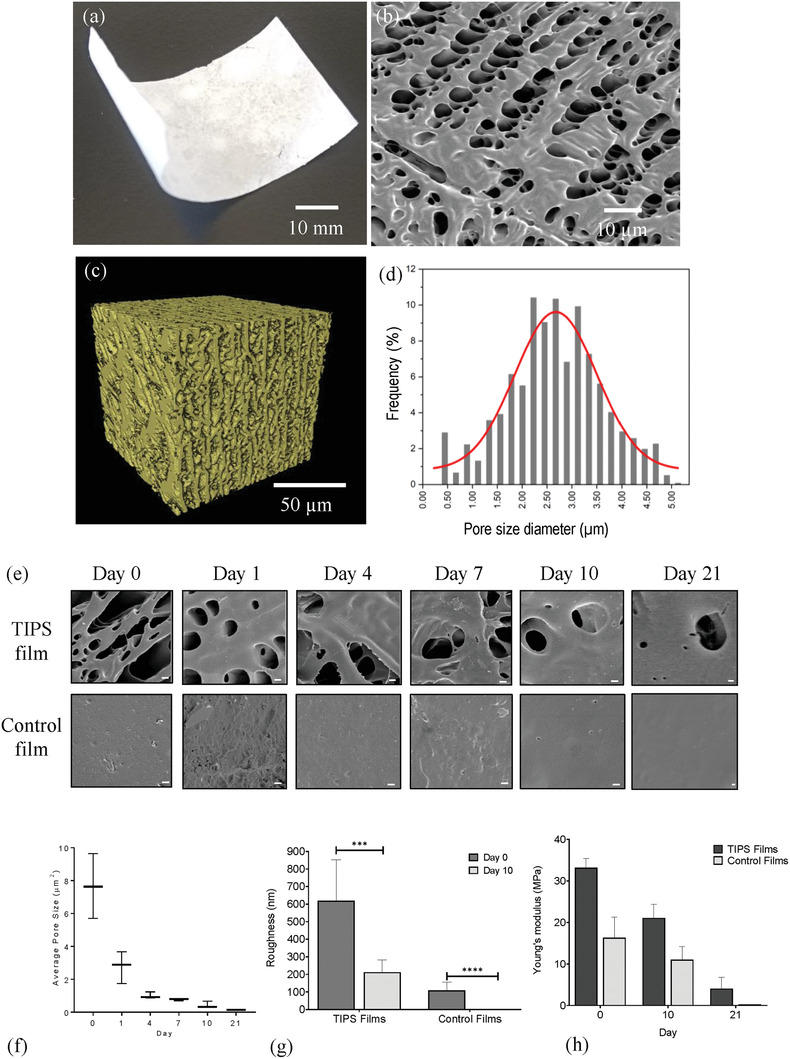
a) Macroscopic image of nonhydrated TIPS film at day 0. b) Scanning electron microscopy (SEM) of TIPS film surface at day 0. c) Volume rendering of TIPS film pore space at day 0 using micro‐CT (calculated porosity 58%). d) The frequency of the pore size (µm) distribution and percentage frequency of pores in the TIPS film at day 0 were measured from the micro‐CT scans. e) SEM images of TIPS and control nonporous films undergoing simulated physiological degradation in vitro at days 0, 1, 4, 7, 10, and 21 showing the change in surface topography as the material degrades via hydrolysis (scale bar: 1 µm). f) Quantification of changes in the area of pores in the TIPS films undergoing simulated physiological degradation in vitro was analyzed in SEM images using ImageJ software. Data were analyzed using one‐way ANOVA with Tukey's multiple comparisons tests. Significant differences were detected between pore area at day 0 and subsequent timepoints (*p* < 0.001). g) Average roughness measurements of TIPS and control nonporous films undergoing simulated physiological degradation in vitro acquired using atomic force microscopy (AFM) at days 0 and 10. Three to five 50 µm × 50 µm areas per sample were imaged at 512 × 512 pixels. From these images, the average roughness values in nanometer were obtained using JPK analysis software source (JPK Instruments, Germany). Data were analyzed using Mann–Whitney test between days 0 and 10 for each type of film (*** *p* < 0.001, **** *p* < 0.0001). h) Young's modulus of TIPS film and control nonporous films undergoing simulated physiological degradation in vitro at days 0, 10, and 21 was calculated using the Oliver–Pharr method from indentation curves measured through nanoindentation. Data were analyzed using two‐way ANOVA with Tukey's multiple comparisons tests. Significant differences were detected within each group for all timepoints (*p* < 0.0001), except between day 0 and day 10 control films. Significant differences were detected between TIPS films and control films at each time point (*p* < 0.0001), except at day 21.

Surface analysis of polymer films using scanning election microscopy (SEM) revealed the TIPS films exhibited an open porous surface with the polymer organized into a network of differently sized ridges and struts, interspersed by interconnected pores (**Figure** **1**b). The porous structure was formed when the temperature of a thin film of polymer solution was lowered by quenching in liquid nitrogen, resulting in de‐mixing of the homogeneous polymer solution into a polymer‐rich and a polymer‐lean phase.^[^ ^36^ ^]^ Subsequent lyophilization of the frozen polymer film removed the frozen solvent, leaving behind the porous polymer structure. Micro‐computed tomography (CT) revealed a microporous structure was present throughout the entire thickness of the films (Figure 1c), with an estimated total porosity of ≈58%. The normal distribution of pore size throughout the film ranged from ≈450 nm to 5 µm, with a median value of 2.676 µm (Figure 1d). The control films used in the study were solid and did not contain any micropores.

A key feature of PLGA that has led to its widespread clinical use is its ability to undergo degradation by hydrolysis, resulting in monomers of lactic and glycolic acids that are metabolized and excreted from the body.^[^ ^37^ ^]^ Simulated physiological degradation of the TIPS polymer films in an aqueous in vitro environment resulted in a loss of surface porosity and roughness compared with the dry starting material (Figure 1e,f), which coincided with a progressive reduction in elastic modulus of the material from 33.2 ± 2.2 to 1.37 ± 2.8 MPa over the 21 days of simulated degradation (Figure 1g). When the TIPS polymer film undergoes simulated degradation in vitro, the polymer becomes softened due to plasticization, as indicated by the reduction in mechanical strength shown in Figure 1g. Previous studies by Blaker et al. have suggested plasticization of the TIPS polymer matrix and associated loss of mechanical properties of the polymer led to distortion of the porous structure of highly porous poly(d,l‐lactide) composite foams at the microlevel resulting in collapse of pore walls and loss of ordered pore structure.^[^ ^38^ ^]^ In the current study, it is likely that a similar mechanism accounts for the progressive loss of pores observed in the TIPS films as they underwent simulated degradation in vitro. A comparison of the mechanical properties of TIPS films versus solid films composed of the same polymer has not previously been reported but previous studies suggest the presence of pores in the polymer films is likely to influence their mechanical properties during degradation. A comparison between PLGA TIPS scaffolds and salt‐leached scaffolds reported differences in mechanical strength, with the thicker polymer walls of the salt‐leached scaffold having reduced mechanical strength attributed to an autocatalytic hydrolysis effect.^[^ ^39^ ^]^

**Figure 2:**
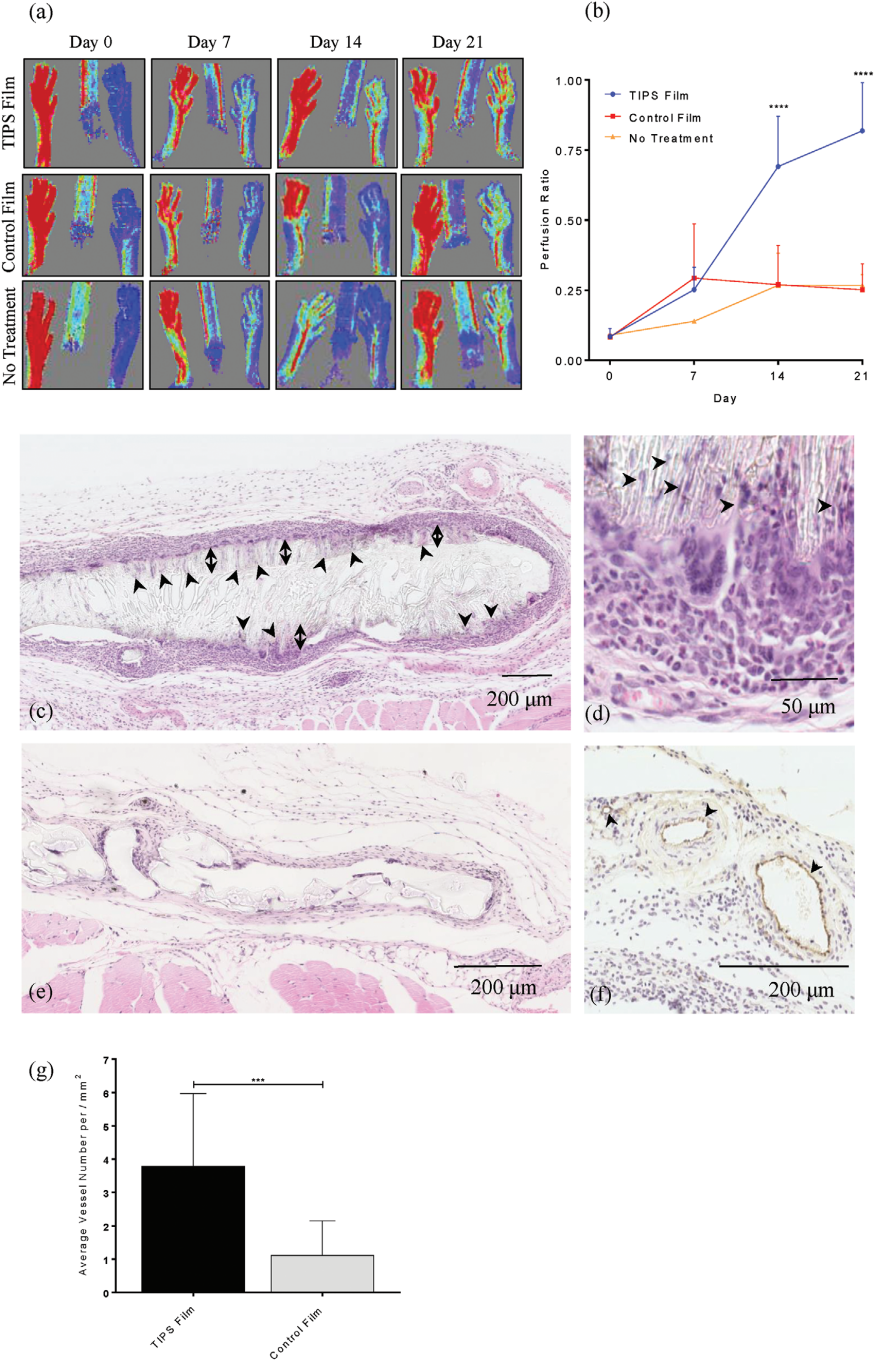
a) Laser Doppler imaging of the paws of mice that had undergone unilateral femoral artery ligation and subsequent implantation of TIPS films, control nonporous films, or received no treatment after ligation. Doppler images were acquired immediately after surgery (day 0) and at days 7, 14, and 21. b) Quantification of laser Doppler imaging of the paws of mice that had undergone unilateral femoral artery ligation and subsequent implantation of TIPS films, control nonporous films or no treatment after ligation. Data are presented as the perfusion ratio at days 7, 14, and 21 relative to the perfusion in the contralateral control limb (*n* = 3 per group). Data were analyzed using two‐way ANOVA with Tukey multiple comparisons testing (**** *p* < 0.0001.) c) Hematoxylin and eosin (H&E) staining of tissue sections collected from the hind limb ischemia model containing the implanted TIPS polymer film at day 21. Cells can be seen infiltrating the porous structure of the TIPS film (arrowheads). A band of cells (denoted by the double‐headed arrow) appears to have migrated into the porous structure of the TIPS film. d) Higher magnification of an area of the TIPS film shows cells (arrowheads) infiltrating aligned channels perpendicular to the surface of the film. A cluster of macrophages at the interface between the film and the host tissue have formed multinucleated giant cells. e) H&E staining of the control nonporous film at day 21. The band of inflammatory cell infiltrate surrounding the implanted material appeared to be less dense compared with the TIPS film. f) Von Willebrand factor (VWF) staining of blood vessels (arrowheads) in tissue surrounding the implanted TIPS film. g) Quantification of the number of blood vessels positively stained for VWF in tissue surrounding the implanted TIPS film and control nonporous film at day 21. The number of vessels was counted by three independent observers. (*n* = 5 mice per group; five fields of view per sample. Data analyzed using Mann–Whitney test; *** *p* < 0.001.)

Following initial in vitro evaluation, the proangiogenic properties of the TIPS polymer films and their ability to restore blood perfusion in ischemic tissue in vivo were compared with control nonporous films composed of the same polymer. Each type of material was overlaid onto the site of vascular occlusion created using an established preclinical model of PAD that results in hind limb ischemia.^[^ ^40^, ^41^, ^42^ ^]^ The level of hind limb blood perfusion following vascular occlusion and treatment, assessed using Laser Doppler imaging at weekly intervals over 21 days, was significantly increased at day 14 and day 21 in the group receiving TIPS polymer films compared with either the group receiving control nonporous polymer films or the group receiving no treatment following vascular occlusion (*p* < 0.0001; **Figure** **2**a,b). At day 14 after ligation, the mean perfusion ratio value for the TIPS polymer film group was 0.691 ± 0.180 compared with 0.270 ± 0.140 in the control nonporous film group and 0.267 ± 0.115 in the no treatment group. At day 21 after vascular occlusion, the mean perfusion ratio value for the TIPS polymer film group was 0.818 ± 0.172 compared with 0.251 ± 0.093 in the control nonporous film group and 0.267 ± 0.038 in the no treatment group. These data indicate that at timepoints beyond 7 days postvascular occlusion, TIPS polymer films have the potential to stimulate angiogenesis and restore vascular perfusion to a level that is close to that of the untreated control limb at day 21. In contrast, vascular perfusion in the group receiving the nonporous control films remained close to that of the group receiving no treatment postvascular occlusion, where the level of perfusion did not increase between day 7 and day 21 postligation.

At day 21, tissues surrounding the implanted materials at the site of vascular occlusion were explanted and analyzed for putative mechanisms that could account for the increased blood flow associated with treatment using TIPS polymer films. Histological analysis of the explanted tissue revealed functional restoration of tissue perfusion corresponded with an increase in blood vessel density in the tissue surrounding the TIPS polymer films compared with the blood vessel density observed in the tissue surrounding the control nonporous polymer films (Figure 2c,f,g). Erythrocytes were visible within the lumen of the vessels indicating that the new vessels were patent and functioning, which would contribute to an increased flux value from moving red blood cells in the vasculature recorded in the Doppler imaging.

The increased vascularization associated with the TIPS polymer films indicates that the TIPS‐fabricated film elicits a particular proangiogenic host response compared with nonporous polymer films composed of the same material. It is therefore reasonable to propose that the physical features of the porous TIPS films account for the differential angiogenic response observed rather than a nonspecific angiogenic response associated with a foreign body reaction due to the implantation of polymer material per se. Multinucleated giant cells were more evident at the interface between the host tissue and the implanted TIPS polymer films (Figure 2d). Fusion of macrophages to form multinucleated giant cells is a phenomenon often associated with materials implanted for longer periods of time.^[^ ^43^ ^]^ The inflammatory cell infiltrate associated with a foreign body reaction arising from degradable materials, such as that observed with the PLGA TIPS films, is transient, self‐limiting, and resolves once the material completely degrades.^[^ ^44^, ^45^ ^]^ This is essential to avoid the onset of chronic inflammation and fibrosis associated for devices intended for therapeutic purposes. The phenotype(s) of the macrophages surrounding the implanted films is likely to be influenced by the structural features of the material and may provide further insight into the mechanisms underlying the functional improvement in vascularization observed. Of particular relevance is the influence of the porous structure of the TIPS films. Porosity has previously been shown to affect macrophage polarization, with cells outside porous implants found to have a predominantly regenerative M2 phenotype associated with secretion of proangiogenic factors.^[^ ^45^ ^]^ Porosity of materials has been identified as a key determinant of angiogenesis in surrounding tissues. For example, nondegradable polytetrafluoroethylene (PTFE) membranes containing pores large enough for host cell penetration (0.8–8 µm) were associated with a higher number of blood vessels compared with the same material exhibiting smaller (0.02 µm) pores.^[^ ^46^ ^]^ Likewise, cardiac implantation of sphere‐templated, poly(2‐hydroxyethyl methacrylate‐*co*‐methacrylic acid) (pHEMA‐*co*‐MAA) hydrogel scaffolds with larger pore diameters (40–80 µm) was associated with an angiogenic response when implanted into the myocardium of immunocompetent rats, which coincided with macrophage polarization toward M2 phenotype.^[^ ^47^ ^]^ Multinucleated giant cell infiltration into the implanted material was only observed with the TIPS polymer films due to the lack of porosity in the control film. Further investigation is required to establish whether particular macrophage phenotype(s) plays a key role in the increased vascularization and tissue perfusion observed and how this is influenced by changes to the physical properties of the material as it degrades over time. It is likely that the angiogenic response to the implanted material arises from a coordinated interaction of multiple cell types within the local environment. This includes not only macrophage subtypes that release key factors involved in angiogenesis and chemotaxis, but also pericytes and endothelial cells required for vascular remodeling, as suggested elsewhere.^[^ ^48^ ^]^

The impact of disease on the angiogenic response observed should also be considered, since the current study used healthy animals with treatment implemented immediately after the onset of ischemia. Ideally models used for further in vivo testing will reflect comorbidities associated with a chronic ischemic state to ascertain whether the angiogenic effect observed is replicated in scenarios that reflect more closely clinical disease.

**Figure 3:**
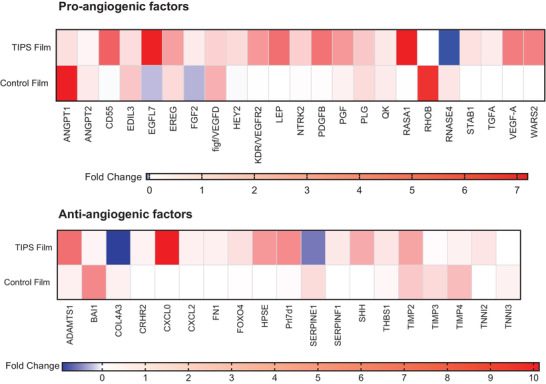
Heat maps illustrating the relative expression of pro‐ and antiangiogenic growth factor genes that exhibited a >0.5‐fold change in expression in tissue collected from the hind limb ischemia model at the site of implantation of the TIPS film or control film at day 21 normalized to nontreatment control.

To identify potential mechanisms that may account for the differences in angiogenesis observed in vivo between the implanted materials, relative levels of angiogenic growth factor gene expression in tissue collected from the implant site were compared from the different experimental conditions. Several proangiogenic genes were upregulated in the explanted tissue containing the TIPS film compared with tissue containing the control nonporous polymer films (**Figure** **3**). These included CD55, Egfl7, epiregulin (Ereg), FGF 2, Hey2, kinase insert domain receptor (KDR; also known as vascular endothelial growth factor receptor 2), leptin (LEP), neurotrophic tyrosine kinase receptor type 2 (Ntrk2), PDGF‐B, placental growth factor (PGF), plasminogen (PLG), QK peptide (QK), RAS p21 protein activator 1(RASA‐1), Stablin (STAB)1, VEGF‐A, and tryptophanyl‐tRNA synthetase, mitochondrial (WARS2), all factors that are associated with controlling key stages of angiogenesis, including blood vessel morphogenesis and vasculogenesis.^[^ ^49^, ^50^, ^51^, ^52^, ^53^, ^54^, ^55^, ^56^, ^57^, ^58^, ^59^, ^60^, ^61^, ^62^, ^63^ ^]^ Conversely, the relative expression of certain genes that control factors that contribute to inhibition of angiogenesis was lower in tissues collected from the TIPS polymer film group compared with the control nonporous polymer film group. These included brain‐specific angiogenesis inhibitor 1 (BAI1), type IV collagen *α*3 (COL4A3), Serpin E1, tissue inhibitor of metalloproteinases (TIMP)‐3, TIMP‐4, and Troponin I3 (TNNI3).^[^ ^64^, ^65^, ^66^, ^67^, ^68^, ^69^ ^]^ While the gene array employed in the current study focused on the expression of angiogenic growth factors, future studies should also pay attention to the role of the host immune response to the implanted material in regulating angiogenesis, which could include extending the gene analysis to the immune‐response mediated by macrophages. The current study lacks a definitive mechanistic connection between pore architecture and increased angiogenesis. Further in vivo studies using appropriate knockout models or agents capable of blocking specific pathways associated with angiogenesis and/or the immune response are necessary to interrogate the mechanistic relationship between porosity and angiogenesis. Future studies will also enable better understanding of the stability and remodeling of newly formed vessels and how this corresponds with degradation of the material overtime.

Nevertheless, the findings in the current study support the concept that materials can be designed to beneficially manipulate the foreign body reaction to achieve a physiological response that can be used to therapeutic effect.^[^ ^70^, ^71^, ^72^ ^]^ Unlike existing biological‐based approaches that involve delivery of individual proteins or genes, either as a single or combined entity, neovascularization observed in the current study results from the activation and secretion of a combination of proangiogenic gene factors from cells that interact with the microporous material. The observed increase in expression of multiple gene‐associated angiogenesis indicates the TIPS polymer film elicits a polygenic response that simulates more closely the natural process of angiogenesis that results in the formation of stable and functional vasculature.

Microporous scaffolds composed of other materials such as bioceramics, decellularized extracellular matrix, and microchanneled 3D‐printed scaffolds have also been associated with a proangiogenic effect.^[^ ^73^, ^74^, ^75^ ^]^ However, in contrast to the current study, these materials have been designed with features primarily intended for other applications, such as accelerating osteogenesis. Moreover, unlike the current investigation, these studies have not reported whether the angiogenesis observed in response to their materials is of therapeutic value and capable of restoration of blood flow in ischemic tissue. The authors are unaware of any previous reports involving the use of similar acellular, nonfunctionalized, porous polymer films that have achieved a similar therapeutic angiogenic response.

Since the PLGA TIPS polymer material degrades over time, further investigation is necessary to establish the duration of the proangiogenic response and whether changes in the stiffness and porosity of the material observed in vitro contribute to the response observed in vivo. Furthermore, since the TIPS process is highly versatile, the method used to produce films in the current study provides scope to produce polymer films that exhibit different roughness and pore structures via adjustment of parameters including the polymer:solvent ratio, composition of PLGA (ratio of lactide to glycolide), solvent, and rate of quenching.^[^ ^27^ ^]^ The ability to control these parameters through further refinement of the porous films provides greater opportunity to fine tune the proangiogenic effect achieved compared with other approaches investigated for therapeutic angiogenesis, such as gene‐based therapy, thus lowering concerns over oncogenic risks. This provides scope for future studies aimed at elucidating optimal properties of the porous polymer films for regulating therapeutic angiogenesis. The stability of the dry, preimplanted TIPS polymer films composed of PLGA, together with the relatively simple manufacturing process, provides an opportunity for low‐cost, “off‐the‐shelf products” that are compatible with long‐term storage. In addition to PAD, TIPS polymer films may have potential use in the treatment of other forms of cardiovascular disease, e.g., cardiac patches for treatment of ischemia or films for dermal wounds and ulcers.

## 3 Conclusion

This study provides compelling evidence to support the concept that microporous TIPS films are capable of stimulating a range of proangiogenic genes in vivo that provide a favorable local environment for neovascularization, increased tissue perfusion, and therapeutic angiogenesis in ischemic tissue. These findings pave the way to establishing new material‐based strategies to achieve therapeutic angiogenesis.

## 4 Experimental Section

### Preparation of TIPS Polymer Films

PLGA 7507 (PURASORB PDLG 7507; 75:25 dl‐lactide/glycolide copolymer; inherent viscosity, 0.70 dL g^−1^, Corbion Biomaterials, Gornchem, Netherlands) was dissolved in dimethyl carbonate (DMC) (anhydrous >99%, Sigma–Aldrich, UK) (10 wt%). Solutions were mixed for 18 h at room temperature. 13 mm diameter 0.16 mm thick D263 M borosilicate cover glasses (VWR, UK) were coated in the polymer solution by holding individual coverslips with forceps and manually submerging them into the polymer solution. Excess solution was displaced by blotting the edge of the coverslip on a paper towel. The polymer‐coated coverslips were immediately horizontally immersed into liquid nitrogen to achieve thermally induced phase separation. The frozen samples were transferred to a −80 °C freezer before being transferred to a freeze–dryer (Edwards Modulyo, Edwards, UK). Once under vacuum, the samples were lyophilized for 18 h to allow the sublimation of residual DMC from within the material.

### Preparation of Control Polymer Films

13 mm diameter borosilicate cover glasses were dip‐coated with PLGA 7507 polymer dissolved in DMC (10 wt%) and placed onto Parafilm to air‐dry over 72 h in a fume hood.

### Hydration of TIPS Polymer and Control Polymer Films

TIPS polymer films and control polymer films were hydrated by placing individual films into the wells of an ultralow attachment 24‐well plate (Corning Costar) containing 0.5 mL ethanol (7% v/v) in phosphate‐buffered saline (PBS). The plate was placed on a plate shaker and incubated at 37 °C with gentle agitation for 12 h. The ethanol solution was removed, and the films were washed in 1 mL PBS prior to further use.

### Simulated Degradation In Vitro

Hydrated TIPS and control polymer films were transferred to sterile ultralow attachment 24‐well plates and incubated in 1 mL PBS at 37 °C for 1, 4, 7, 10, and 21 days. The degraded samples were dried in a desiccator prior to ultrastructural analysis. PBS was used for the degradation study in accordance with media used in standardized degradation studies (ISO 10993–9).

### Ultrastructural Imaging of the Polymer Films

SEM was used to examine ultrastructural features of the polymer films degraded under simulated physiological conditions. The samples were mounted onto aluminum stubs using carbon sticky tabs and sputter‐coated with gold/palladium alloy in an argon atmosphere. Samples were imaged using a Jeol 7401 high‐resolution field emission SEM. The area of the pores in the degraded TIPS films was measured from SEM images (*n* = 3) using image analysis software (ImageJ 1.52a).

X‐ray micro‐CT was used to analyze the internal structure of the TIPS films using a ZEISS Xradia 520 Versa micro‐CT system (Carl Zeiss X‐ray Microscopy Inc., Pleasanton, CA, USA). A 5 mm circular segment of the TIPS film was collected using a disposable biopsy punch. An area of 510 µm × 510 µm of the TIPS film was scanned acquiring 1401 projections and 57 s exposure time with camera binning set at 1. The X‐ray tube was operated at 40 kV and 3 W, employing the 20× objective lens in order to achieve a voxel size of 223 nm.

A filtered back projection algorithm (FDK) was used to reconstruct the radiographs into a 3D volume using the Zeiss proprietary image reconstruction software package (Zeiss MReconstructor, Carl Zeiss X‐ray Microscopy Inc., Pleasanton, CA, USA).

Data visualization and segmentation were performed with Avizo software (Thermo Fisher Scientific, France). A 500 × 500 × 500 voxel (111 × 111 × 111 µm) volume was extracted in order to avoid artifacts at the edge of the scan and to maximize the contrast between pore and polymer phases during the segmentation process. The grayscale images were subsequently segmented into a binary dataset assigning pixels to the pore phase and to the polymer film by the way of a thresholding procedure. FiJi software package was used on the binarized datasets for computing local thickness values and average pore size distribution.^[^ ^76^ ^]^

### Surface Texture Analysis of the Polymer Films

Atomic force microscopy (AFM) was used to measure the surface roughness of the polymer films undergoing in vitro simulated physiological hydrolytic degradation. The polymer films were hydrated before incubation in 1 mL PBS at 37 °C for 1, 4, 7, and 10 days. Surface analysis of the hydrated (or dry control) films was performed using a Bruker Nanowizard IV atomic force microscope (Bruker, Santa Barbara, CA, USA) fitted with a Respa‐10 etched silicone probe or an MSNL‐10 6 cantilever 0.01–0.5 N m^−1^ sharp nitride level probe (Bruker, Coventry, UK). An area covering 50 µm × 50 µm was measured at a resolution of 512 × 512 pixels, with 3–5 scans per sample. The average roughness (*R*_a_) value was calculated (JPK analysis software; JPK Instruments, Germany) prior to any image processing.

### Stiffness of the Polymer Films

The mechanical properties of the polymer films undergoing in vitro simulated physiological hydrolytic degradation were characterized using an Anton Paar Bioindenter (UNHT³ Bio; Anton Paar, Austria) fitted with a Berkovich diamond tip. The polymer films (dry control films or films hydrated in PBS) were fixed onto a polystyrene Petri dish, and measurements were conducted at room temperature. Four indentation measurements were performed on each sample at intervals of 50 µm apart. A loading rate of 30 mN min^−1^ with a maximum loading rate of 5 mN was used with a 5 s hold. The elastic modulus was calculated from indentation curves using the Oliver–Pharr method.^[^ ^77^ ^]^

### In Vivo Assessment of Polymer Films in Ischemic Tissue

Circular samples (5 mm Ø) of the polymer films for implantation were created using a biopsy punch. The films were hydrated, washed with 1 mL PBS and placed into one PBS prior to implantation. Hind limb ischemia was induced in female 2–3 month old c57bl/6 mice by unilateral femoral artery ligation.^[^ ^78^ ^]^ Briefly, under general anesthesia (O_2_ flow meter at 1 L min^−1^ and isoflurane to 1.5%), an incision along the center of the medial left thigh was made using fine scissors. The common femoral artery was exposed and isolated from the femoral vein and nerve. The artery was then ligated with a triple suture, proximally to the deep femoral artery bifurcation. Immediately following artery ligation, the polymer films were placed over the occluded vessel bundle. Bupivacaine analgesia was administered intramuscularly before the wound was closed. Vessel occlusion was confirmed by laser Doppler imaging (moorVMS‐LDF, Moor Instruments, UK) immediately after surgery, with further measurements of tissue perfusion recorded at weekly intervals to day 21. Tissue perfusion in the ischemic limb was normalized against the control contralateral limb of each animal. At day 21 following surgery, the mice were euthanized by overdose of CO_2_, followed by cervical dislocation, and the leg muscles from the test site and contralateral control limb were harvested for analysis. All experiments were performed under a UK Home Office license (PLN: 70/7700), in compliance with the 1986 United Kingdom Home Office Animals (Scientific Procedures) Act and with the approval of the University College London local ethics committee.

Qiagen RT^2^ Angiogenic Growth Factors Array (Qiagen, UK) was used to profile gene expression of angiogenic‐related growth factors from tissue surrounding the implanted TIPS film or control polymer film, or tissue collected from nontreated controls. About 10–30 mg of tissue was mechanically homogenized and RNA extracted using Monarch Total RNA MiniPrep Kit (Monarch, UK) in accordance with the manufacturer instructions. RNA concentration and purity were measured using a NanoDrop 8000 UV Visible Spectrophotometer (ThermoScientific, UK). To create complementary DNA (cDNA) from the extracted RNA and eliminate genomic DNA, the RNA was reverse‐transcribed using QuantiTect Reverse Transcription Kit (Qiagen, UK). cDNA was prepared for the RT^2^ PCR Array 96‐well plate using RT^2^ SYBR Green ROX qPCR Mastermix and the array analyzed using the Aligent Mx3005p qPCR system, with the thermal profile set to run one cycle at 95 °C for 10 min, 95 °C for 15 s, 55 °C for 35 s, and 72 °C for 30 s with 40 cycles. Fold change was calculated by first normalizing TIPS film and control film (TE) quantification cycle (*C*_q_) values from their respective housekeeping gene values (HE) (TE − HE = ΔCTE). Nontreatment control (TC) *C*_q_ values were normalized from housekeeping control values (HC) (TC − HC = ΔCTC). The difference between the test and nontreatment control *C*_q_ values was calculated (ΔCTE − ΔCTC = ΔΔ*C*_t_) and expressed fold change was determined (2 − ΔΔ*C*_t_).

### Histological Analysis

Leg muscle tissues were fixed in neutral buffered formalin solution (4%) and processed into Paraplast X‐TRA low‐temperature paraffin wax. Tissue sections were stained with hematoxylin and eosin (H&E) or immunostained for using anti‐Von Willebrand factor (VWF) antibody (Ab11713, Abcam, UK) to identify blood vessels in the tissue sections. The stained tissue sections were imaged using a Nanozoomer 2.0‐HT (Hamamatsu, UK) digital slide scanner. The number of positively stained blood was counted in 1 mm^2^ areas of tissue taken from five fields of view in each tissue section. Manual vessel counting was performed individually by three blinded observers.

### Statistical Analysis

Data were analyzed using the GraphPad Prism (version 8.0), as indicated in the figure legends. Mean values were plotted with standard deviation. One‐way analysis of variance (ANOVA) or two‐way ANOVA with Tukey multiple comparisons testing was used to compare differences between two or more groups. Mann–Whitney test was used to analyze differences between two independent nonparametric data sets.

## Conflict of Interest

The authors declare no conflict of interest.
